# Editorial: Conflicts and humanitarian crises on displaced people's health

**DOI:** 10.3389/fpubh.2023.1234576

**Published:** 2023-07-11

**Authors:** Ahmed Hossain, Andrea Bartolucci, Shela Akbar Ali Hirani

**Affiliations:** ^1^College of Health Sciences, University of Sharjah, Sharjah, United Arab Emirates; ^2^Institute of Security and Global Affairs (ISGA), Leiden University, Leiden, Netherlands; ^3^Faculty of Nursing, University of Regina, Regina, SK, Canada

**Keywords:** displaced peoples, humanitarian crises, health, refugees, conflicts

In the last Global trends report, the UNHCR estimates that global forced displacement has topped 100 million people, with roughly 50 million children among them (1). There are around 32.5 million refugees worldwide, with developing countries hosting 85% of them; only Syrian Arab Republic, Venezuela, Afghanistan, South Sudan, and Myanmar account for more than two-thirds of all refugees relocated abroad (1). Conflicts and humanitarian crisis negatively affect all aspects of health and wellbeing of the displaced people, and expose them to torture, abuse and a variety of communicable and non-communicable diseases (2). Many disparities exit in the distribution of the humanitarian aid to displaced population (3). Displaced people have long been denied access to basic healthcare, and hostilities in many locations are worsening an already poor situation. Furthermore, rebuilding a country's healthcare system to a pre-conflict level can take years, jeopardizing health outcomes.

Hence, it is indisputable that a dedicated edition in Frontiers in Public Health that explore the factors that enhance the health of displaced individuals is warranted. The Research Topic titled “*Conflicts and humanitarian crises on displaced people's health*” delves into significant topics pertaining to the favorable elements that guide individuals toward their health. The published articles represent contributions that analyze the abilities, skills, aptitudes, and experiences conducive to improving healthcare across various displaced populations worldwide. The present Research Topic highlights several significant contributions, which are outlined as follows.

Hirani et al. examined healthcare professionals' COVID-19 pandemic experiences in refugee settings (3). Such workers have risked viral infection, fatigue, and mental illness (Khan et al.). The study also analyzes humanitarian COVID-19 frontline healthcare professionals' self-preparedness using constructivist grounded theory. The study indicated that Bangladeshi frontline healthcare professionals self-prepared in three phases: (a) **Pandemic shock**, where workers were astonished by the pandemic and worried about their health and that of their patients; (b) **Pandemic awareness**; where workers learned about the virus and how to avoid it and they also developed pandemic-stress coping techniques; and (c) **Pandemic resilience**, because, despite the pandemic, workers were able to work and they formed a community with their coworkers and supported each other.

Ivasiy et al. reported that Russia's invasion of Ukraine interrupted healthcare services, particularly opioid agonist treatment (OAT) programs. OAT program directors have had to react to war disruptions like widespread internal displacement and legislative revisions, providing more flexibility with OAT distribution rules and take-home dose regulations. Eight directors from seven Ukrainian regions were interviewed in depth about their experiences providing OAT during the war and the local crisis-response approach under the emergency policy updates. The directors' experiences were categorized by geography and patient displacement. OAT programs also struggled due to patient migration across the nation. Many patients had to transfer to areas without OAT services. This study's OAT directors were able to continue serving patients despite problems. Their experiences show how OAT programs can be adapted to provide care during conflict.

Jebril et al. investigated the relationship between war-related trauma and blood pressure trajectory in Gaza among mid-aged and older persons. War-related trauma, such as injury, family death, or house bombing, was linked to elevated blood pressure in the study. The study also indicated that prolonged war-related trauma increased the likelihood of high blood pressure. Finally, this study suggests that war-related traumatic events can affect blood pressure and that reducing exposure to them could prevent hypertension.

In Cox's Bazar, Bangladesh, Qayum et al. surveyed FDMNs and the host population about oral cholera vaccination (OCV) coverage. The October 2022 survey randomly selected 1,200 households. The host community had 72% OCV coverage, whereas FDMNs had 84%. Children aged 1–5 (89%) had higher coverage than adults aged 15–64 (74%). The survey revealed that Cox's Bazar's low cholera rate might be due to high OCV coverage. Figure 1 shows a Myanmar woman who forced to relocate to Bangladesh expressed her disillusionment with regard to her pursuit of an improved quality of life.

**Figure 1:**
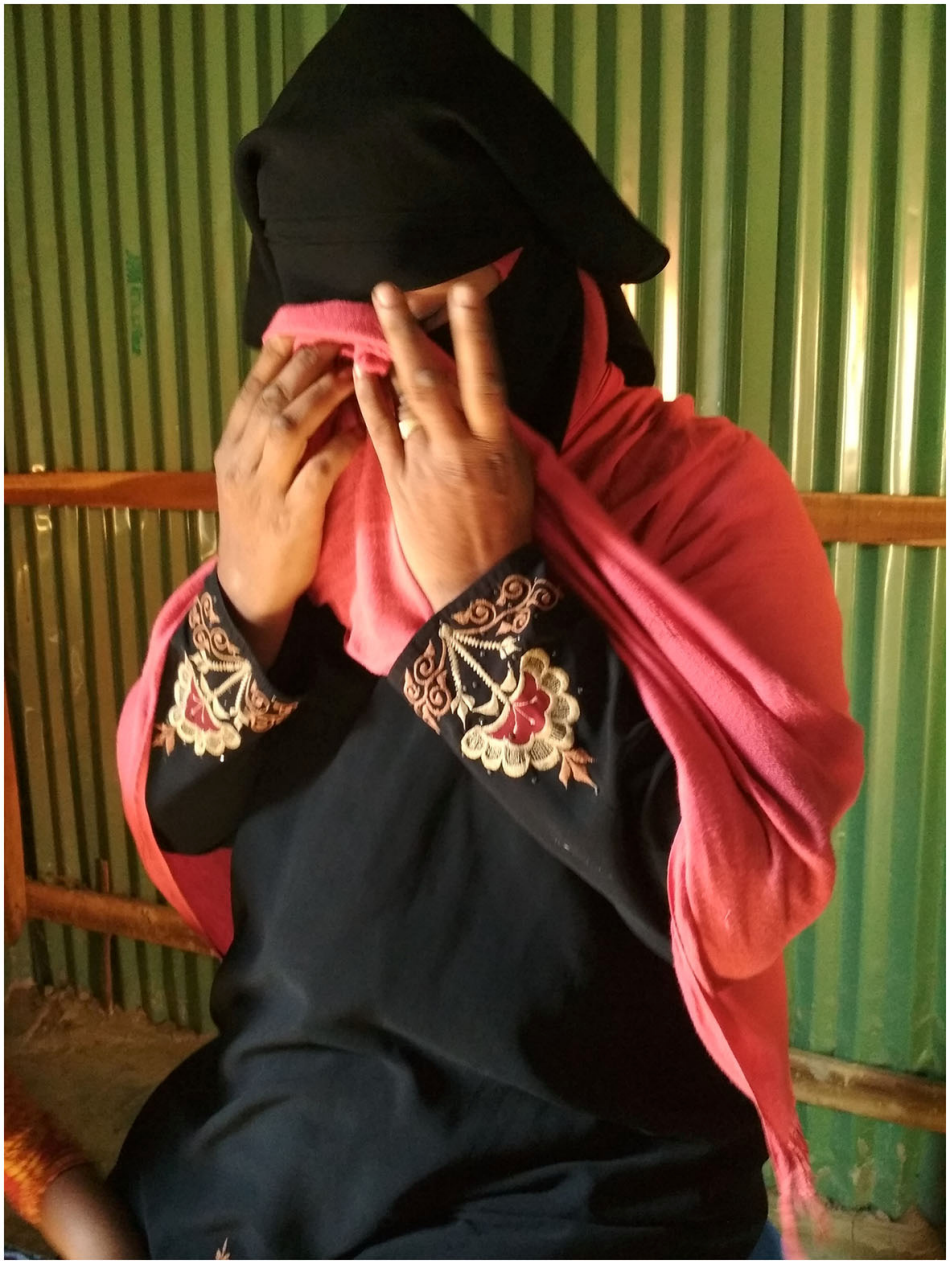
A Myanmar woman who forced to relocate to Bangladesh expressed her disillusionment with regard to her pursuit of an improved standard of living.

Lastly, Eneh et al. discussed the 2016 Syrian cholera outbreak. The Syrian humanitarian crisis has caused widespread displacement and a clean water and sanitation shortage, worsening the outbreak. Syria had ~500,000 cholera infections and 10,000 deaths by March 2023. The paper stresses the need for a unified Syrian cholera response. The government, humanitarian organizations, and the commercial sector must respond. Preventing future epidemics requires a sustained response.

## Author contributions

The initial draft of the article was written by AH, with subsequent contributions made by AB and SH to enhance its quality. The final manuscript was reviewed and approved by all authors.
